# Peptide hydrogel boosts the cytotoxic and metabolic fitness of Vγ9Vδ2 T cells in melanoma immunotherapy

**DOI:** 10.3389/fimmu.2026.1793631

**Published:** 2026-03-20

**Authors:** Hongjie Liu, Man Li, Ying Jiao, Lilumeng Huang, Qi Liu, Shuang Xiao, Mingjie Zhan, Xianfeng Zha, Li Shi, Zhinan Yin, Zheng Xiang, Chengbiao Yang, Yan Xu

**Affiliations:** 1 The Affiliated Guangdong Second Provincial General Hospital of Jinan University, Guangzhou, Guangdong, China; 2 State Key Laboratory of Bioactive Molecules and Druggability Assessment, The Biomedical Translational Research Institute, Health Science Center (School of Medicine), Jinan University, Guangzhou, Guangdong, China; 3 Department of Clinical Laboratory, The First Affiliated Hospital, Jinan University, Guangzhou, Guangdong, China; 4 Fourth Affiliated Hospital of Guangzhou Medical University, Guangzhou, Guangdong, China; 5 Department of Microbiology and Immunology, Health Science Center (School of Medicine), Jinan University, Guangzhou, Guangdong, China; 6 Xiehe Tianjin Overseas Students’ Entrepreneurship Park, Tianjin, China

**Keywords:** immunotherapy, integrin signaling, melanoma, metabolic reprogramming, peptide hydrogel, Vγ9Vδ2 T cells

## Abstract

**Background:**

Adoptive cell therapy with Vγ9Vδ2 T cells represents a promising approach for melanoma treatment. However, its efficacy is often limited by poor persistence, inadequate tumor infiltration, and functional suppression within the tumor microenvironment. Peptide-based hydrogel as a vehicle has exhibited great potential for delivery of biologics and enhancement of their function, but their ability to directly modulate the metabolic and cytotoxic fitness of Vγ9Vδ2 T cells remains largely unexplored.

**Methods:**

We developed a peptide hydrogel (Self-assembly material based on peptide Nap-GFFF, named as SAM.1) and assessed its ability to activate Vγ9Vδ2 T-cell and amplify their cytotoxicity to A375 melanoma cells *in vitro*, and to enhance antitumor efficacy in a melanoma xenograft model. Mechanistic studies focused on integrin signaling, PI3K/AKT/mTOR activation, and metabolic reprogramming.

**Results:**

SAM.1 significantly enhanced the cytotoxic activity of Vγ9Vδ2 T cells against A375 melanoma cells *in vitro*. It promoted Vγ9Vδ2-T cell activation, evidenced by increased CD25 and CD69 expression, and boosted the secretion of key cytotoxic effector molecules such as IFN-γ, TNF-α and perforin. Mechanistically, SAM.1 engaged integrin signaling (upregulating CD11c and CD103), leading to activation of the PI3K/AKT/mTOR pathway. This signaling cascade drove a beneficial metabolic reprogramming, shifting T cell energy production from glycolysis towards oxidative phosphorylation, thereby enhancing their bioenergetic capacity. Beyond that, peritumoral delivery of Vγ9Vδ2 T cells increased intratumoral T cell infiltration. As a result, melanoma growth was inhibited after administration of SAM.1 encapsulating Vγ9Vδ2 T cells.

**Conclusion:**

SAM.1 hydrogel acted as a two-in-one scaffold, controlled release and an immunomodulatory agent, to enhance the persistence and antitumor function of Vγ9Vδ2 T cells. This strategy provided a new paradigm for γδ T-cell–based immunotherapy in melanoma.

## Introduction

Melanoma is one of the most aggressive and therapy-resistant malignancies of the skin and mucosa, characterized by rapid progression, early metastasis, and high mortality (1, 2). Global epidemiological studies have indicated that the incidence and mortality of melanoma continue to rise, with new cases expected to exceed half a million annually by 2040 (3). Despite significant progress in immune checkpoint blockade (ICB) and adoptive cell therapies, clinical outcomes remain heterogeneous. While anti-PD-1/PD-L1 and anti-CTLA-4 antibodies have achieved durable responses in subsets of patients, many still develop resistance or experience immune-related toxicities (4–8). Thus, new therapeutic strategies are urgently needed to improve treatment efficacy and overcome resistance in melanoma.

Among emerging immunotherapies, γδ T cells have gained attention due to their unique biology and potent cytotoxicity against tumors (9, 10). Unlike conventional αβ T cells, γδ T cells recognize stress-induced ligands and phosphoantigens independently of MHC restriction, enabling rapid and broad-spectrum antitumor responses (11–14). In particular, Vγ9Vδ2 T cells—the predominant subset in human peripheral blood—exert strong cytotoxic effects through perforin/granzyme release, Fas/FasL-mediated apoptosis, and the secretion of pro-inflammatory cytokines such as IFN-γ and TNF-α (15–17). Clinical studies have correlated intratumoral γδ T cell infiltration with favorable prognosis in cancer patients (18). Nevertheless, the clinical application of Vγ9Vδ2 T cells faces major challenges, including limited persistence, insufficient tumor infiltration, and suppression within the tumor microenvironment (TME) (19, 20). Strategies such as phosphoantigen stimulation (e.g., zoledronic acid, pamidronate) combined with IL-2 or ex vivo-expanded autologous/allogeneic γδ T cells have demonstrated feasibility in hematologic and solid tumors (21–28), yet their therapeutic efficacy in melanoma remains suboptimal.

Biomaterial-based strategies present promising opportunities to enhance the efficacy of adoptive cell therapies. Previous studies have shown that hydrogels can be engineered to deliver T cells or cytokines locally, improving their persistence and reshaping the TME (29–32). However, the clinical application has been limited seriously due to cumbersome manufacture, addition of various cytokines, and biosafety risk, etc. Due to good manufacturing practices, biocompatibility and designable bioactivity, peptides have been broadly studied as building blocks to construct hydrogel for small molecular drug delivery (33–36), cell cultures (37–39) and regenerative medicine (40, 41), etc (42–44). However, hydrogel based peptides as bio-scaffold for cell delivery protection, and local retention has not been investigated in depth, including the potential of peptide-engineered hydrogels to augment the cytotoxic and metabolic functions of Vγ9Vδ2 T cells for the therapy of melanoma.

In this study, we designed a peptide hydrogel based on self-assembling sequences, using 2-Naphthylacetic acid (Nap) and D-Phenylalanine (F) due to their excellent ability to self-assemble and enhance the immune response against antigen. We synthesized three peptide sequences—Nap-FF, Nap-GFF, and Nap-GFFF—and assessed their ability to self-assembly, with Nap-GFFF (SAM.1) showing the best self-assembly characteristics. We hypothesized that a hydrogel constructed by a specific peptide motif, such as Nap-GFFF, would not only release the γδT cell sustainly, but also activate them for improving the tumor immunotherapy. This hypothesis was supported by our findings that Nap-GFFF could boost the cytotoxic and metabolic fitness of Vγ9Vδ2 T cells for melanoma immunotherapy. We hypothesized that a hydrogel functionalized with a specific peptide motif would release T cells, maintain and activate T cells and reprogram T cell metabolism towards a robust effector phenotype, and ultimately, tumor-killing capacity. We systematically evaluated the effects of this matrix on the expansion, metabolic profile (including oxidative phosphorylation and glycolysis), cytokine secretion, and *in vitro* and *in vivo* cytotoxic efficacy of Vγ9Vδ2 T cells against melanoma models. Our findings demonstrate that this versatile peptide hydrogel acts as a potent artificial niche, significantly augmenting the antitumor response and tumor tissue recruitment of Vγ9Vδ2 T cells, thereby presenting a novel and effective combinatorial strategy for solid tumor immunotherapy.

## Materials and methods

### Expansion of Vγ9Vδ2 T cells from PBMCs of healthy donors

Experiments were performed using buffy coats derived from healthy volunteer whole blood donations (supplied by the blood bank of the Red Cross, Zhuhai, China). Zoledronic acid (Zol) expanded of Vγ9Vδ2 T cells were generated as described (45). Briefly, peripheral blood mononuclear cells (PBMCs) were isolated by Ficoll-Paque density gradient centrifugation (GE Healthcare, USA). Vγ9Vδ2 T cells were expanded in RPMI-1640 medium (Gibco, USA) containing 10% fetal bovine serum (FBS). To activate cells, Zol (Sigma, Germany) was added at a final concentration of 50 μM at day 0. 100 IU/mL recombinant human IL-2 (Beijing Sihuan Biotech, China), 100 IU/mL IL-15 (PeproTech, USA) and 70 μM vitamin C (Sigma, Germany) were added every 2 days from day 3. After 10–14 days of culture, the purity of the expanded cells was determined by flow cytometry using CD3-FITC (BD Biosciences,clone:UCHT1) and Vδ2-PE (BioLegend,clone:B6), was consistently >90%.

### Preparation and characterization of peptide hydrogels

Three self-assembling materials—SAM.1 (Nap-GFFF), SAM.2 (Nap-FF), and SAM.3 (Nap-GFF)—used in this study were synthesized by GL Biochem (China). For hydrogel preparation, 2 mg of each peptide was transferred into a 1.5 mL glass vial and dissolved in 700 μL of sterile phosphate-buffered saline (PBS, pH 7.0). The pH of the solution was carefully adjusted to 7.0 using a sodium carbonate solution, and the final volume was brought to 1000 μL with additional PBS. The mixture was then heated to boiling to ensure complete dissolution of the peptide. After cooling to room temperature, the self-assembling materials based short-peptide were acquired and subsequently stored at 4°C. For *in vitro* cell stimulation assays, aliquots of the hydrogel stock were diluted into complete RPMI 1640 medium to achieve the indicated final working concentrations (typically 5–50 μg/mL). At these diluted concentrations, the material does not form a self-supporting macroscopic gel but exists as dispersed peptide assemblies/nanofibers suspended in the culture medium. Cells were therefore exposed to the material by direct mixing in the medium, without surface coating or cell encapsulation.

### Flow cytometry analysis

Flow cytometry was used to evaluate Vγ9Vδ2 T cell activation, cytokine production, apoptosis, and integrin expression. Briefly, cells were stained with antibodies CD3-Spark Blue 550 (BioLegend,clone:OKT3), Vδ2-FITC (BioLegend,clone:B6), CD69-BV480 (BD Biosciences,clone:FN50), CD25-PE-Fire 640 (BioLegend,clone:M-A251), CD107a-PE (BioLegend,clone:H4A3), and CD137-APC (BioLegend,clone:4B4-1). Lymphocytes were first gated based on forward and side scatter properties, followed by singlet discrimination and live cell gating. Vγ9Vδ2 T cells were defined as CD3^+^Vδ2^+^ cells, within which subsequent analyses were performed. For intracellular cytokine staining, cells were stimulated with PMA (50 ng/mL), ionomycin (1 μg/mL), and GolgiPlug (BD Biosciences, USA) for 4–6 h. After staining for surface markers, the cells were fixed and permeabilized, using Lysing Solution (BD Biosciences, USA) and Permeabilizing Solution (BD Biosciences, USA), respectively. Subsequently, the cells were stained with antibodies IFN-γ-PE-Cy7 (BD Biosciences,clone:B27), TNF-α-PerCP-Cy5.5(eBioscience,clone:MAb11), and Perforin-BV421 (BD Biosciences,clone:dG9). Apoptosis was assessed with Annexin V–FITC/7-AAD staining (BioLegend, USA). For integrin profiling, cells were stained with fluorochrome-conjugated antibodies against integrin αX (CD11c-Alexa Fluor 647,clone:3.9) and integrin αE (CD103-APC-Cy7,clone:Ber-ACT8). Fluorescence minus one (FMO) controls were used to establish positive gating thresholds for all multicolor flow cytometry analyses. Representative gating strategies and FMO controls are provided in the Supplementary Information (**Supplementary Figure 1**). All samples were acquired on a Cytek Aurora flow cytometer (Cytek Biosciences, USA) and analyzed using FlowJo software (BD Biosciences, USA).

### Cytotoxicity assay

A flow cytometry-based lysis assay was performed in order to determine the killing activity of γδ T cells against the tumor cell lines A375 melanoma cells. Target cells were labeled with CFSE (2 μM; Invitrogen, USA) and co-cultured with effector T cells at effector-to-target (E:T) ratios of 0:1 and 10:1 at 37°C for 6 h. The cell apoptosis of cancer cells was analyzed using flow cytometry and PI (0.2 µg/mL;BD Biosciences, USA) staining. Dead target cells were identified as CFSE^+^PI^+^ by flow cytometry. Cytotoxicity was calculated as: Cytotoxicity (%) =[(% of dead target cells–% of spontaneous death)/(100– % of spontaneous death)] × 100%.

### Seahorse metabolic flux analysis

The metabolic profile of Vγ9Vδ2 T cells was assessed using a Seahorse XF Analyzer (Agilent, USA). Oxygen consumption rate (OCR) and extracellular acidification rate (ECAR) were measured in mitochondrial and glycolysis stress tests. Sequential injections included oligomycin (1 μM), FCCP (1 μM), rotenone (1 μM), and antimycin A (1 μM) for OCR, or glucose (10 mM), oligomycin (1 μM), and 2-deoxy-D-glucose (2-DG, 50 mM) for ECAR. Data were analyzed with Wave software (Agilent, USA).Seahorse measurements were performed using cells from independent human donors, with 1–2 technical wells per condition per donor; donor numbers are reported in the corresponding figure legends.

### Western blotting

Cells were lysed in RIPA buffer containing protease and phosphatase inhibitors. Equal protein samples were separated by SDS-PAGE and transferred onto PVDF membranes (Millipore, Germany). After blocking with 5% BSA, membranes were incubated with primary antibodies against PI3K, AKT, p-AKT, mTOR, p-mTOR, 4E-BP1, and S6K (Cell Signaling Technology, USA). After washing, membranes were incubated with HRP-conjugated secondary antibodies, and signals were visualized with enhanced chemiluminescence (Bio-Rad,USA).

### Signaling inhibition assays

To elucidate the underlying signaling mechanisms, Vγ9Vδ2 T cells were pretreated with rapamycin (10 nM; MedChemExpress, USA) for 15min before hydrogel stimulation to inhibit mTOR signaling, which is known to occur rapidly at the post-translational level. For integrin blockade, soluble RGD peptide (50 μM; Selleck, USA) was added 3h prior to hydrogel treatment to allow sufficient inhibition of integrin-mediated adhesion and modulation of surface integrin expression (CD11c and CD103). Following treatment, cells were subsequently analyzed by flow cytometry.

### Murine melanoma xenograft model

All animal studies were approved and performed in compliance with the guidelines for the use of experimental animals by the Institutional Animal Care and Use Committee (IACUC) of South China Agricultural University. Four-week-old male B-NDG mice (20 ± 2 g) were purchased from Biocytogen (Zhuhai, China) and housed under specific pathogen–free conditions at the Animal Center of South China Agricultural University.A375 human melanoma cells (2×10^6^/mouse) were injected subcutaneously into the right flank of B-NDG mice. Once tumors reached 40–60 mm³, mice were stratified according to tumor volume to ensure comparable baseline tumor sizes, and then randomly assigned to one of four treatment groups (n = 5 mice per group): (1) Control, (2) Vγ9Vδ2 T cells (s.c.), (3) SAM.1 + Vγ9Vδ2 T cells (i.v.), (4) SAM.1 + Vγ9Vδ2 T cells (s.c.). Mice were randomly assigned to treatment groups following stratification by baseline tumor volume. Blinding was not performed during treatment administration or tumor measurement. Human Vγ9Vδ2 T cells were administered at a dose of 1 × 10^7^ cells per mouse. Cells were either injected alone or encapsulated within SAM.1 peptide-based hydrogel (50 μg/mL, 100 μL per injection). According to group assignment, treatments were delivered via subcutaneous peritumoral injection (s.c.) adjacent to the tumor mass or via intravenous injection (i.v.) through the tail vein. Control mice received an equal volume of sterile saline. All treatments were administered once weekly for four consecutive weeks. Tumor size was measured every 2–3 days, and tumor volume was calculated as: V = (length × width²)/2. When the tumor volume exceeds 2,000 mm³, the mice will be euthanized and counted as dying. The tumors were excised for qPCR and flow cytometry analysis of Vγ9Vδ2 T cell infiltration, and serum cytokines were measured by BioLegend LEGENDplex™ Human Inflammation Panel 1 (13-plex) with Filter Plate(Cat#740808) (BioLegend, USA).

### Quantitative real-time PCR analysis of Vγ9Vδ2 T cell infiltration

Tumor tissues were collected before and 4 days after treatment, snap-frozen in liquid nitrogen, and stored at −80°C. Total RNA was extracted using the RNA Easy Fast Animal Tissue/Cell Total RNA Extraction Kit (Tiangen, China), and cDNA was synthesized after genomic DNA removal. Quantitative PCR was performed with TB Green Premix Ex Taq II (Takara, Japan) following the manufacturer’s protocol. Gene expression levels were normalized to β-actin and calculated using the 2^–ΔCt^ method, with results expressed as the mean of three replicates.

### Statistical analysis

Data are expressed as mean ± SEM. Statistical analyses were performed using GraphPad Prism 9.0 (GraphPad Software, USA). Tumor growth curves were analyzed using a two-factor mixed-effects model (REML) with treatment group and time as fixed effects and mouse as a random effect, which appropriately accounts for repeated longitudinal measurements within each animal. Where appropriate, *post hoc* multiple comparisons between groups at individual time points were performed using Tukey’s correction. Comparisons between two groups were analyzed using Student’s t-test, as appropriate. Statistical significance was defined as P < 0.05.

## Results

### Physicochemical characterization, biocompatibility, and controlled release of peptide hydrogels

**Figure 1:**
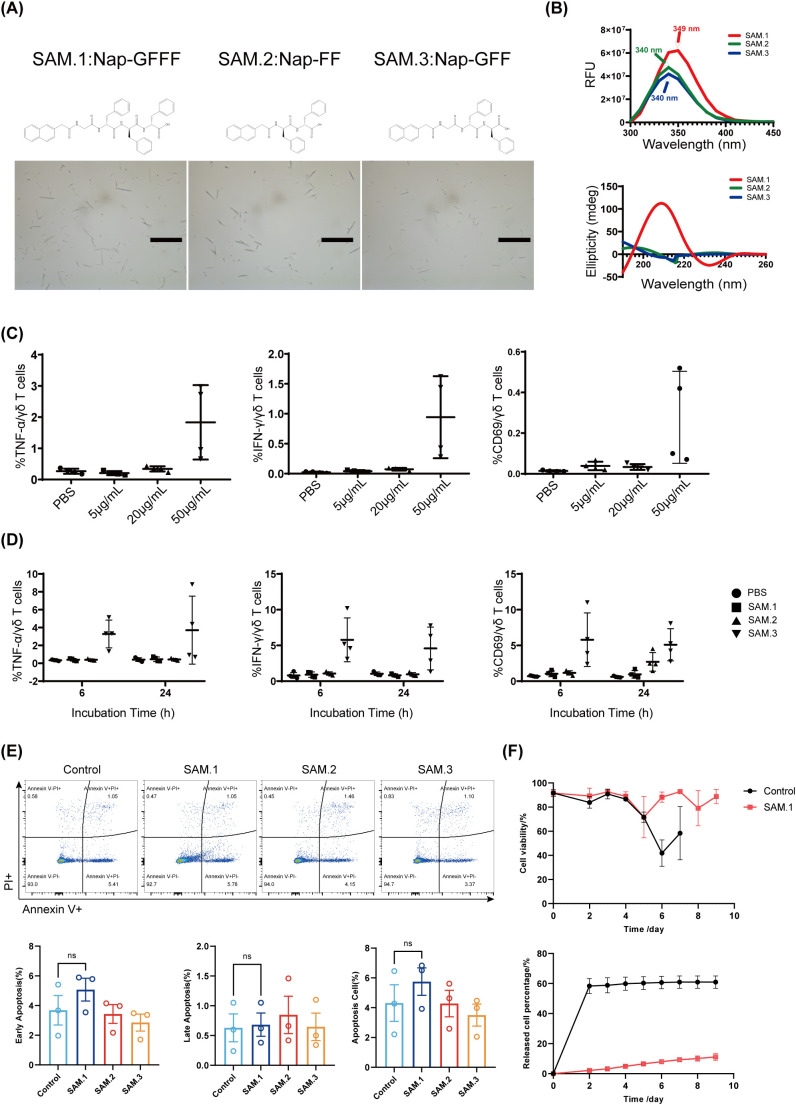
Structural characterization and cytocompatibility of peptide hydrogels. **(A)** Chemical structures of three peptide hydrogels (SAM.1, SAM.2, SAM.3) and their self-assembled morphologies observed by optical microscopy. **(B)** Fluorescence emission spectra and circular dichroism (CD) profiles of the hydrogels, highlighting composition-dependent photophysical and chiral properties. **(C)** Dose–response analysis of SAM.1 at increasing concentrations (0–50 μg/mL) on Vγ9Vδ2 T cell activation, measured by TNF-α, IFN-γ, and CD69 expression. **(D)** Comparison of 6 h versus 24 h incubation with 50 μg/mL SAM.1, showing differential effects on Vγ9Vδ2 T cell activation and cytokine production. **(E)** Annexin V/PI staining and flow cytometric quantification of apoptosis in Vγ9Vδ2 T cells after 6 h co-culture with the indicated hydrogels. **(F)** Transwell-based controlled release of Vγ9Vδ2 T cells from SAM.1 hydrogel and viability of released cells over time. Data are shown as mean ± SEM from independent human donors. For panels **(C, D)**, n = 4 donors; for panels **(E, F)**, n = 3 donors. ns, P > 0.05.

In this study, 2-Naphthylacetic acid (Nap) and D-Phenylalanine (F) were chosen due to their excellent ability of self-assembly and immune activation. We used Nap and F as unit to design three peptides, Nap-FF, Nap-GFF and Nap-GFFF, respectively. Beyond Nap-GFF, other both could self-assemble to hydrogel at the concentration of 2 mg/mL after heating-cooling cycle. We first characterized the microstructure of the three peptide-based materials (Nap-GFFF as SAM.1, Nap-FF as SAM.2, and Nap-GFF as SAM.3). All three resulting self-assembly materials were nanofibers (**Figure 1A**). Then, the capability of self-assembly was evaluated through fluorescence emission and circular dichroism (CD). SAM.1 showed a maximum emission at 349 nm, whereas SAM.2 and SAM.3 displayed peaks at 340 nm (**Figure 1B**), which indicated that Nap-GFFF possessed better ability to self-assembly. According to CD spectra, the peak of SAM.1 was at 190 nm and 208 nm, indicating that formed beta-sheet structure (**Figure 1B**). And the SAM.2 and SAM.3 tend to self-assemble to be alfa-helix.

Then the bioactivity of three self-assembly materials were examined. We aimed to determine the optimal peptide sequence, concentration and incubation duration for the application *in vitro*. Firstly, Vγ9Vδ2 T cells were co-incubated with SAM.1 at varying concentrations (0 μg/mL, 5 μg/mL, 20 μg/mL, and 50 μg/mL) for 6 hours. The effects on T-cell activation and cytokine secretion were subsequently evaluated. The results demonstrated that treatment with 50 μg/mL SAM.1 led to a markedly higher expression of the activation marker CD69 compared with the other concentration groups. In addition, the secretion levels of the cytotoxic cytokines TNF-α and IFN-γ were highest at this concentration (**Figure 1C**). These findings indicated that 50 μg/mL SAM.1 hydrogel most effectively activates Vγ9Vδ2 T cells. Based on these observations, a concentration of 50 μg/mL was selected for subsequent experiments in this study. After that, we further investigated the effect of incubation time and peptide sequence on the functional activity of Vγ9Vδ2 T cells. Cells were co-cultured with SAM.1, SAM.2 and SAM.3 (50 μg/mL) for 6 h or 24 h respectively, followed by assessment of CD69, TNF-α, and IFN-γ expression. The results showed that the expression of all three markers were significantly upregulated after treatment by SAM.1 compared with SAM.2 and SAM.3 (**Figure 1D**). However, both incubation duration were not significantly different. These observation suggested that SAM.1 based peptide Nap-GFFF optimally activated the Vγ9Vδ2 T cells, and persistent stimulation did not impact the activation of Vγ9Vδ2 T cells. Considering both functional efficacy and experimental feasibility, a 6-hour incubation period was selected for subsequent *in vitro* experiments.

To assess biocompatibility, Vγ9Vδ2 T cells were incubated with each SAM for 6 h and analyzed by Annexin V/PI flow cytometry (**Figure 1E**). The proportion of viable cells was similar among all groups (Control 95.27 ± 2.25%, SAM.1 93.93 ± 1.72%, SAM.2 95.40 ± 1.64%, SAM.3 96.13 ± 1.56%), with no significant differences in early apoptosis, late apoptosis, or total apoptosis (all P > 0.05). In a Transwell system, SAM.1 provided controlled release of encapsulated Vγ9Vδ2 T cells over time while maintaining cell viability, consistent with a gentle, cytocompatible matrix environment (**Figure 1F**).

### SAM.1 enhances the cytotoxicity and effector functions of Vγ9Vδ2 T cells *in vitro*

**Figure 2:**
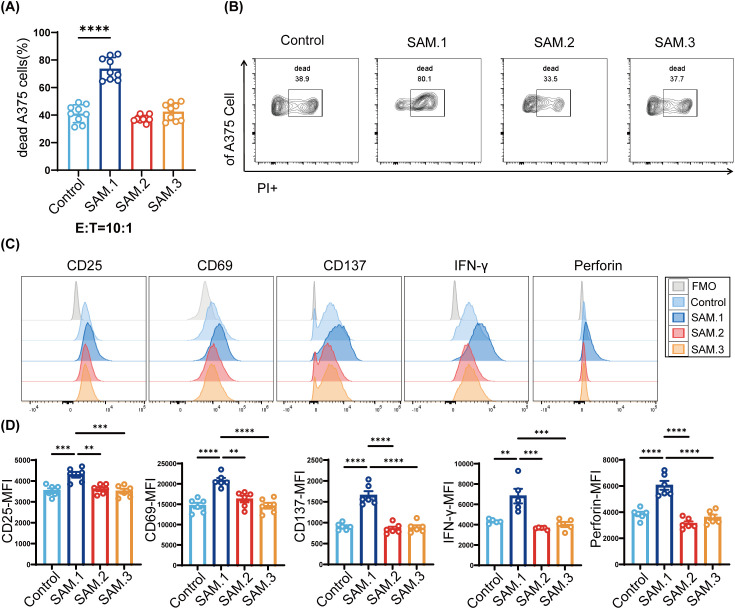
SAM.1 hydrogel enhances the cytotoxicity and effector functions of Vg9Vd2 T cells *in vitro*. **(A, B)** Cytotoxicity of Vγ9Vδ2 T cells against CFSE-labeled A375 melanoma cells assessed by flow cytometry after 6 h co-culture (E:T = 10:1) with the indicated peptide hydrogels. Gating strategies were established using fluorescence minus one (FMO) controls. SAM.1 markedly increased tumor cell death compared with Control, whereas SAM.2 and SAM.3 showed no significant enhancement. **(C, D)** Flow cytometry analysis of activation markers (CD25, CD69, CD137) and effector molecules (IFN-γ, TNF-α, perforin) in Vγ9Vδ2 T cells following hydrogels stimulation. SAM.1 significantly upregulated CD25, CD69 and CD137, and increased IFN-γ and perforin (with no comparable changes in SAM.2/SAM.3). Data are shown as mean ± SEM from independent human donors. For **(A–D)**, n = 3 donors (Technical replicates per donor: **A, B**, 3 replicates; **C, D**, 3–4 replicates). **P < 0.01; ***P < 0.001; ****P < 0.0001.

To evaluate whether SAM could magnify the cytotoxic activity of Vγ9Vδ2 T cells, CFSE-labeled A375 melanoma cells were co-cultured with SAM-treated Vγ9Vδ2 T cells at an effector-to-target (E:T) ratio of 10:1 for 6 h. We found that Vγ9Vδ2 T cells incubated by SAM.1 significantly increased tumor cell killing (73.7 ± 8.12%) compared with the control group (41.0 ± 6.46%, P < 0.05), SAM.2 (37.35 ± 2.40%) and SAM.3 (42.25 ± 6.41%) (**Figures 2A, B**). These results demonstrated that SAM.1 significantly enhanced the cytotoxicity of Vγ9Vδ2 T cells against melanoma cells.

We next assessed whether SAM.1 also promoted T-cell activation and effector molecule secretion. Flow cytometry revealed that SAM.1 significantly increased CD25 expression (4,298 ± 138.3 MFI vs. 3,538 ± 109.9 MFI in controls, P < 0.001), CD69 (20,824 ± 640.9 MFI vs. 14,649 ± 692.1 MFI, P < 0.0001), and CD137 (1,656 ± 99.06 MFI vs. 905.5 ± 32.71 MFI, P < 0.0001). Importantly, SAM.1-treated Vγ9Vδ2 T cells displayed elevated IFN-γ (6,814 ± 714.4 MFI vs. 4,330 ± 91.27 MFI, P < 0.01) and perforin (6,048 ± 339.3 MFI vs. 3,857 ± 167.8 MFI, P < 0.0001). In contrast, SAM.2 and SAM.3 hydrogels did not significantly alter activation marker or cytokine expression (**Figures 2C, D**). Collectively, these results indicate that SAM.1 hydrogel not only boosts cytotoxicity but also enhances the activation and effector program of Vγ9Vδ2 T cells *in vitro*.

### SAM.1 promotes oxidative phosphorylation and reduces glycolysis in Vγ9Vδ2 T cells

**Figure 3:**
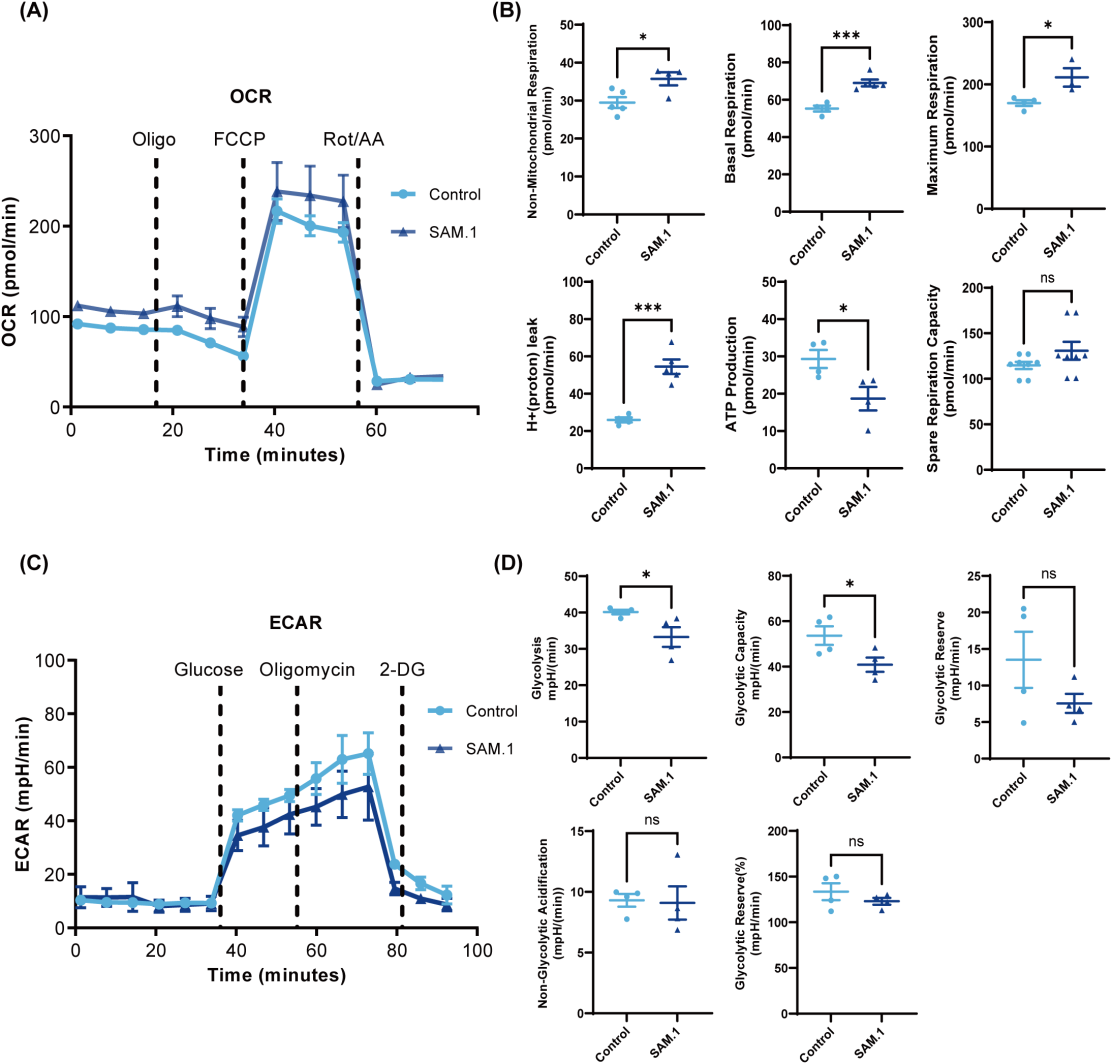
SAM.1 hydrogel promotes mitochondrial oxidative metabolism in Vγ9Vδ2 T cells. **(A)** Oxygen consumption rate (OCR) of Vγ9Vδ2 T cells measured using the Seahorse XF mitochondrial stress test following stimulation with the indicated peptide hydrogels. Oligomycin, FCCP, and rotenone/antimycin A were injected sequentially as indicated. OCR values were normalized to cell number. **(B)** Quantification of OCR-derived parameters, including basal respiration, maximal respiration, and spare respiratory capacity. **(C)** Extracellular acidification rate (ECAR) of Vγ9Vδ2 T cells measured using the Seahorse XF glycolysis stress test. Glucose, oligomycin, and 2-deoxy-D-glucose (2-DG) were injected sequentially as indicated. ECAR values were normalized to cell number. **(D)** Quantification of ECAR-derived parameters, including glycolysis, glycolytic capacity, and glycolytic reserve. Data are shown as mean ± SEM from independent human donors. For **(A–D)**, n = 4 donors. ns, P > 0.05; *P < 0.05; ***P < 0.001.

Upon activation, T cells undergo profound metabolic reprogramming to meet the increased bioenergetic and biosynthetic demands required for proliferation, differentiation, and effector function. Specifically, effector T cells typically transition toward aerobic glycolysis, whereas long-lived and highly functional T cells rely more on mitochondrial oxidative phosphorylation (OXPHOS) to sustain antitumor activity and persistence (46, 47). Therefore, we investigated the effects of SAM.1 on the metabolic profile of Vγ9Vδ2 T cells, including mitochondrial respiration and glycolysis. Vγ9Vδ2 T cells treated with SAM.1 for 6 hours were compared with untreated cells. The metabolic profiling were recorded using a Seahorse XF analyzer, with oxygen consumption rate (OCR) and extracellular acidification rate (ECAR) measured as indicators of oxidative phosphorylation and glycolysis, respectively. As showing in **Figure 3A**, SAM.1 treatment significantly increased OCR, indicative of enhanced OXPHOS. Basal respiration was higher in the SAM.1 group compared with Control (69.05 ± 4.07 vs. 55.29 ± 3.17 pmol/min, P < 0.001), and maximum respiration was also elevated (211.30 ± 25.68 vs. 170.00 ± 9.38 pmol/min, P < 0.05), suggesting that SAM.1 improves mitochondrial function and confers greater spare respiratory capacity (**Figure 3B**). In contrast, glycolysis stress testing revealed that SAM.1 hydrogel reduced ECAR. SAM.1-treated Vγ9Vδ2 T cells exhibited significantly lower glycolysis (33.25 ± 5.41 vs. 40.14 ± 1.24 mpH/min, P < 0.05) and glycolytic capacity (40.81 ± 6.27 vs. 53.66 ± 8.23 mpH/min, P < 0.05) relative to Control cells (**Figures 3C, D**). Collectively, these findings indicate that SAM.1 hydrogel reprograms the metabolism of Vγ9Vδ2 T cells by shifting energy production from glycolysis to mitochondrial respiration. This metabolic shift enhances cellular energy supply, supports functional persistence, and counteracts the Warburg effect, ultimately improving the antitumor efficacy of Vγ9Vδ2 T cells.

### SAM.1 improves both antitumor activity and tumor infiltration of Vγ9Vδ2 T cells *in vivo*

**Figure 4:**
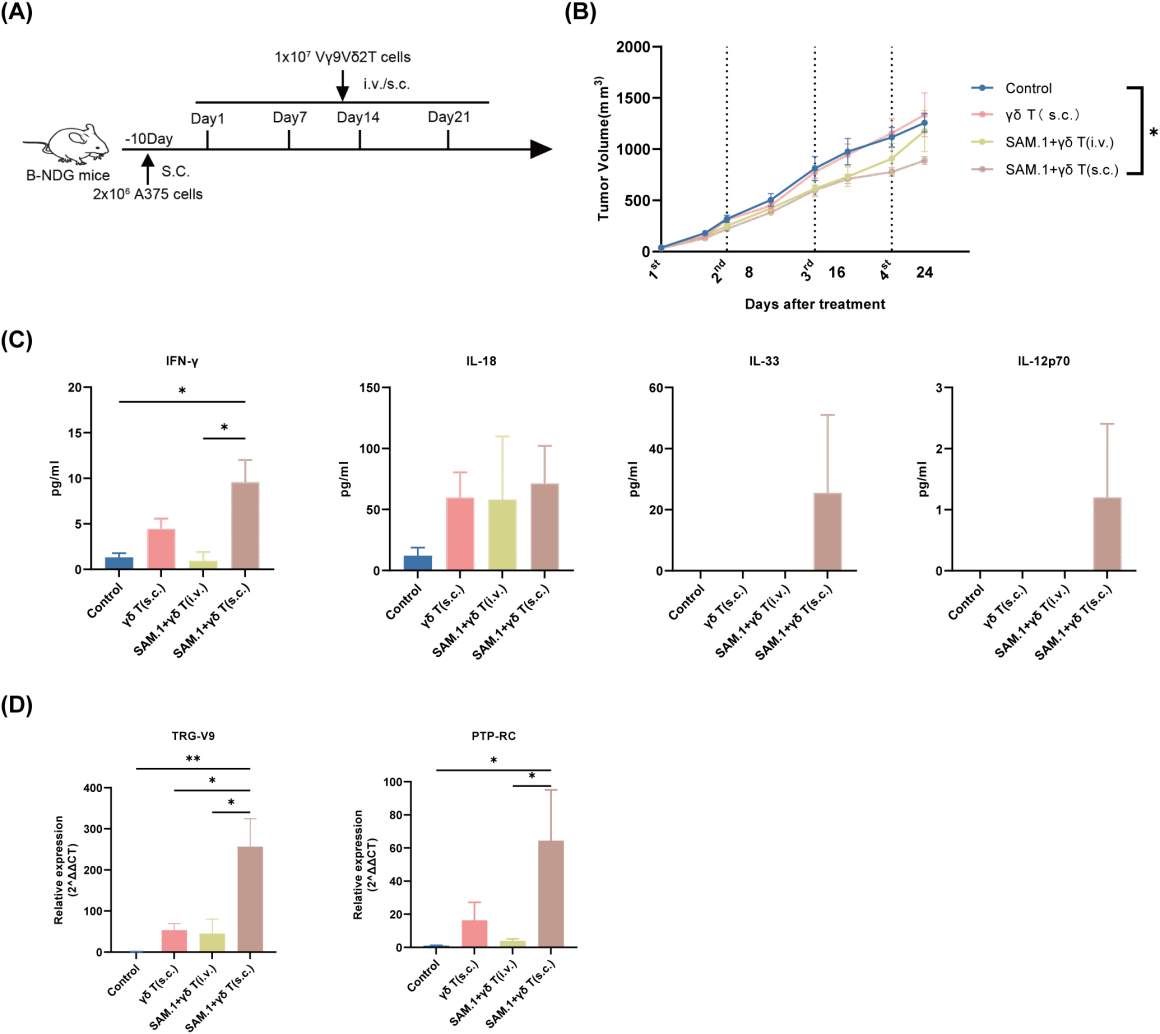
SAM.1 hydrogel augments *in vivo* efficacy and tumor infiltration of Vγ9Vδ2 T cells. **(A)** Schematic of the A375 melanoma xenograft model in B-NDG mice and treatment schedule/groups (Control, Vγ9Vδ2 T cells (s.c.), SAM.1 + Vγ9Vδ2 T cells (i.v.), and SAM.1 + Vγ9Vδ2 T cells (s.c.)). **(B)** Tumor growth curves showing that peritumoral administration of SAM.1-encapsulated Vγ9Vδ2 T cells (s.c.) reduced melanoma growth compared with Control. **(C)** Serum cytokine levels measured on day 3 after cell infusion, showing elevated IFN-γ and increased IL-18, IL-33, and IL-12p70 in the SAM.1 + Vγ9Vδ2 T cells (s.c.) group. **(D)** qPCR analysis of tumor tissues collected on day 4 after infusion showing increased human TRG-V9 and PTPRC (CD45) expression in the SAM.1 + Vγ9Vδ2 T cells (s.c.) group, indicating enhanced intratumoral retention/accumulation of transferred human cells. Data are shown as mean ± SEM from mice. For **(A–D)**, n = 5 mice per group. *P < 0.05; **P < 0.01.

The results *in vitro* demonstrated that SAM.1 enhanced the cytotoxic activity of Vγ9Vδ2 T cells against melanoma cells. Here, we used B-NDG mice inoculated with the human melanoma cell line A375. When the tumor volume reached 40–60 mm³, treatment was initiated according to the predefined protocol (**Figure 4A**). Human Vγ9Vδ2 T cells (1 × 10^7^ cells per mouse) were either administered alone or encapsulated within SAM.1 peptide-based hydrogel (50 μg/mL, 100 μL per injection) and delivered via subcutaneous peritumoral injection (s.c.) adjacent to the tumor mass or via intravenous injection (i.v.) through the tail vein. Tumor growth curves are shown in **Figure 4B**. Mixed-effects analysis revealed a significant effect of time (P < 0.0001), whereas the group × time interaction did not reach statistical significance (P = 0.0868), indicating that overall growth trajectories were not significantly different between groups across the study period. *Post hoc* multiple comparisons suggested reduced tumor volume in the SAM.1 + Vγ9Vδ2 T (s.c.) group relative to control at late time points (e.g., day 24; adjusted P < 0.05).

To further investigate the mechanisms by which SAM.1 hydrogel regulates the *in vivo* function of Vγ9Vδ2 T cells, we first assessed post-treatment immune activation by examining changes in inflammatory cytokines. On day 3 after cell infusion, peripheral blood was collected from mice in each group, and serum was isolated to measure cytokine levels. The results showed that the concentration of IFN-γ in the SAM.1+γδ T (s.c.) group (9.59 ± 4.82 pg/mL) was significantly higher than that in the control group (1.32 ± 0.90 pg/mL, P < 0.01). In addition, the levels of the pro-inflammatory cytokines IL-18, IL-33, and IL-12p70 were also elevated in this group compared with the other groups (**Figure 4C**). Because experiments were conducted in severely immunodeficient B-NDG mice and cytokines were measured using a human-specific LEGENDplex™ assay, the detected cytokines represent human cytokines derived from the infused Vγ9Vδ2 T cells rather than murine host cytokines. The coordinated upregulation of these cytokines suggests that SAM.1 hydrogel may enhance antitumor immune responses by reshaping the systemic immune microenvironment and establishing a proinflammatory positive feedback loop. To further assess the impact of the SAM.1 peptide-based hydrogel on Vγ9Vδ2 T cell tumor infiltration, tumor tissues were collected on day 4 after Vγ9Vδ2 T cell infusion. RNA was extracted from the tumors, and qPCR was performed to evaluate the expression of human-specific Vγ9Vδ2 T cell genes, TRG-V9 and PTPRC. TRG-V9 encodes a variable region of the γ chain of the T cell receptor (TCR), typically forming a heterodimer with the Vδ2 region in Vγ9Vδ2 T cells, whereas PTPRC (also known as PTPRC or CD45) encodes a receptor-type protein tyrosine phosphatase broadly expressed on the surface of immune cells. The qPCR results showed that the mRNA levels of TRG-V9 (256.50 ± 166.1) and PTPRC (64.44 ± 52.95) in the SAM.1+γδ T (s.c.) group were significantly higher than those in the other groups (both P < 0.01 vs. control) (**Figure 4D**). Given the species specificity of these markers, this approach provides a sensitive and quantitative assessment of human Vγ9Vδ2 T-cell presence within murine tumor tissues. These findings indicate that using SAM.1 peptide-based hydrogel as a delivery vehicle enables prolonged retention of Vγ9Vδ2 T cells within the tumor microenvironment, thereby enhancing their antitumor efficacy *in vivo* (**Figure 4D**).

### SAM.1 activates the PI3K/AKT/mTOR pathway to enhance Vγ9Vδ2 T-cell effector function

**Figure 5:**
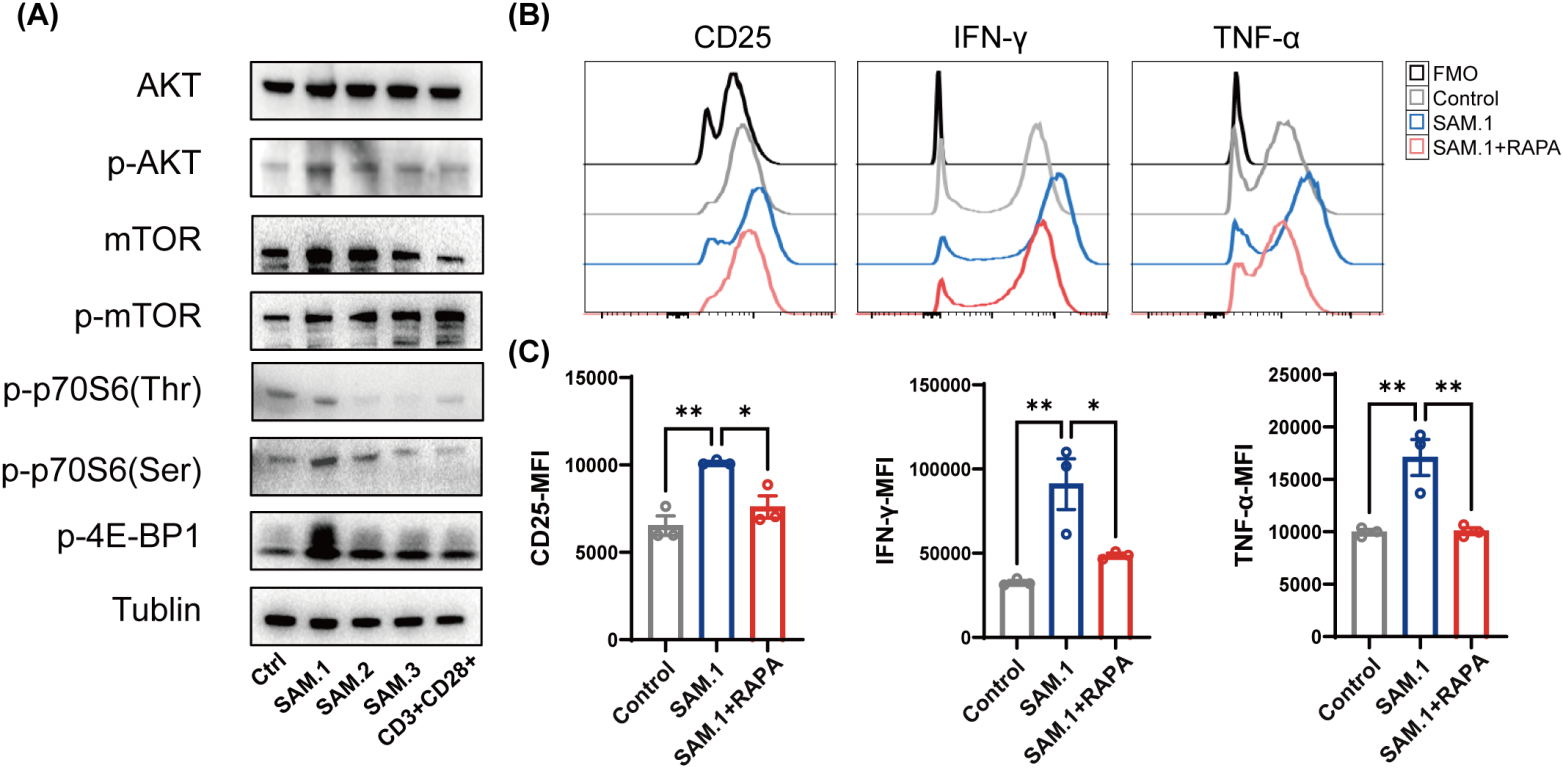
SAM.1 hydrogel activates the PI3K/AKT/mTOR pathway in Vγ9Vδ2 T cells. **(A)** Western blot analysis of PI3K/AKT/mTOR pathway activation in Vγ9Vδ2 T cells after 15 min stimulation with the indicated SAMs. SAM.1 induced increased phosphorylation of AKT and mTOR, and enhanced phosphorylation of downstream targets p70S6K (Ser371) and 4E-BP1 (Thr37/46), compared with Control/SAM.2/SAM.3. **(B, C)** Flow cytometry analysis of CD25 and effector cytokines (IFN-γ and TNF-α) in Vγ9Vδ2 T cells stimulated with SAM.1 with or without rapamycin pretreatment (mTORC1 inhibitor; 10 nM, 15 min). Rapamycin significantly attenuated SAM.1-induced T-cell activation and cytokine production. Data are shown as mean ± SEM from independent human donors. For **(B, C)**, n = 3 donors. *P < 0.05; **P < 0.01.

To elucidate the molecular mechanisms underlying the functional enhancement of Vγ9Vδ2 T cells by SAM.1, we examined the activation of the PI3K/AKT/mTOR signaling axis, a key pathway regulating T-cell growth, metabolism, and effector responses. Vγ9Vδ2 T cells were incubated with different SAMs for 15 min, and expression of pathway components was assessed by Western blot. Compared with Control, SAM2 and SAM3, SAM.1 markedly increased phosphorylation of AKT, mTOR, p70S6K(Ser371), and 4E-BP1(Thr37/46), with the most pronounced upregulation observed for p-4E-BP1. (**Figure 5A**). These results indicate that SAM.1 activated the AKT/mTOR signaling pathway, which leaded to amplification of the antitumor function of Vγ9Vδ2 T cells.

Given our previous findings that SAM.1 hydrogel reprograms Vγ9Vδ2 T-cell metabolism by shifting energy production from glycolysis toward mitochondrial respiration—thereby enhancing bioenergetic capacity, supporting functional durability, and counteracting the Warburg effect—it was essential to determine whether this metabolic adaptation is mechanistically linked to AKT/mTOR activation. To further validate the role of mTOR signaling, cells were pretreated with rapamycin (10 nM), a specific mTORC1 inhibitor, prior to SAM.1 hydrogel exposure. We then assessed the functional consequences of mTOR inhibition on T-cell activation. However, rapamycin pretreatment markedly reduced the expression of CD25 (7,597 ± 1,107 MFI, P < 0.05), IFN-γ (48,707 ± 2,254 MFI, P < 0.05), and TNF-α (10,067 ± 558.5 MFI, P < 0.01) compared with SAM.1 alone (**Figures 5B, C**). Together, these findings demonstrated that SAM.1 excited the PI3K/AKT/mTOR signaling cascade, which resulted in Vγ9Vδ2 T-cell activation and effector cytokine production.

### SAM.1 enhances Vγ9Vδ2 T-cell function through integrin-associated signaling

**Figure 6:**
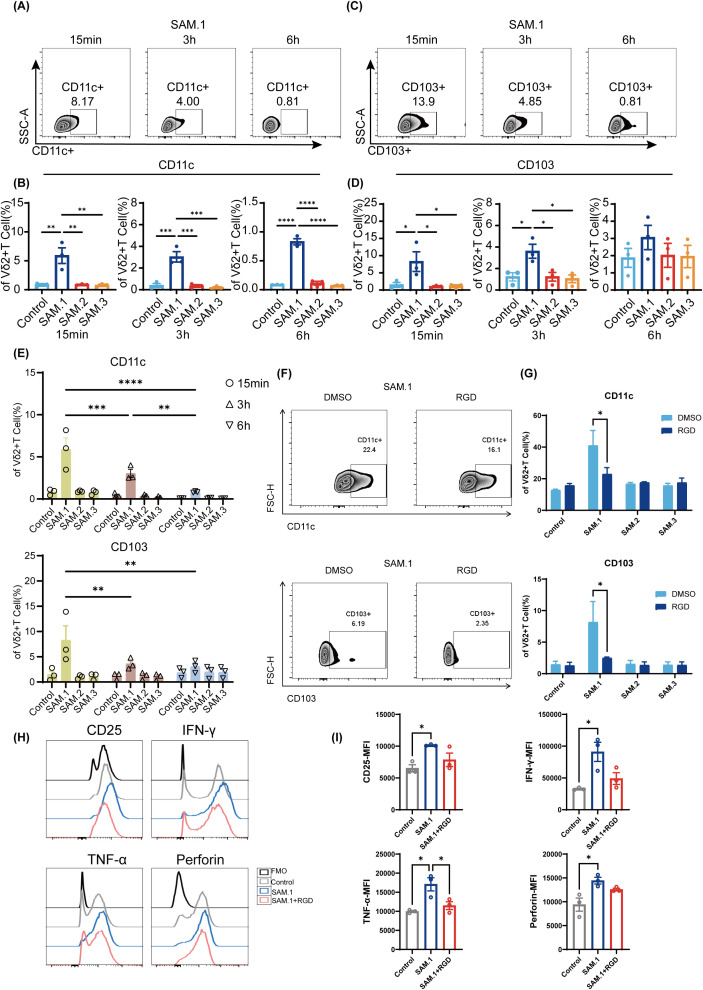
Integrin signaling mediates SAM.1 hydrogel–induced enhancement of Vγ9Vδ2 T-cell function. **(A, B)** Representative flow cytometry plots and quantification of CD11c (integrin αX) expression on Vγ9Vδ2 T cells following stimulation with the indicated peptide hydrogels. **(C, D)** Representative flow cytometry plots and quantification of CD103 (integrin αE) expression on Vγ9Vδ2 T cells following stimulation with the indicated peptide hydrogels. **(E)** Time-course analysis showing that CD11c and CD103 expression on SAM.1-stimulated Vγ9Vδ2 T cells decreased over time. **(F, G)** Flow cytometry analysis of activation-associated readouts in Vγ9Vδ2 T cells stimulated with SAM.1 with or without RGD pretreatment (50 μM, 24 h), showing that integrin blockade attenuates SAM.1-mediated functional enhancement, with no comparable effects observed in Control, SAM.2, or SAM.3. **(H, I)** Intracellular staining of IFN-γ, TNF-α, and perforin in Vγ9Vδ2 T cells after SAM stimulation with or without RGD pretreatment (50 μM, 3h), demonstrating that RGD reduces SAM.1-induced effector molecule upregulation. Data are shown as mean ± SEM from independent human donors. For **(A–I)**, n = 3 donors. *P < 0.05; **P < 0.01; ***P < 0.001; ****P < 0.0001.

The observed metabolic reprogramming suggested that upstream cues might originate from interactions between SAM.1 and surface receptors. Given that integrins are key mediators of mechanotransduction and link extracellular matrix (ECM) cues to intracellular signaling pathways, we explored whether integrin-associated signaling is involved in SAM.1-induced functional enhancement of Vγ9Vδ2 T cells (**Figure 6**). To assess integrin involvement, we analyzed the surface expression of CD11c (αXβ2) and CD103 (αEβ7) on Vγ9Vδ2 T cells following SAM.1 stimulation by flow cytometry. SAM.1 markedly upregulated both integrins, reaching peak levels at 15 min (8.21- and 8.29-fold increases, respectively, vs. Control; P < 0.05) and gradually declining thereafter. Nevertheless, CD11c and CD103 expression remained significantly higher in SAM.1-treated cells than in Controls or SAM.2/SAM.3 groups at all time points tested (15 min, 3 h, 6 h; P < 0.05) (**Figures 6A–E**). These results indicate that SAM.1 rapidly induces integrin upregulation, consistent with the involvement of integrin-associated signaling, which may contribute to downstream PI3K/AKT/mTOR activation. To further examine whether SAM.1-mediated functional enhancement is functionally linked to RGD-sensitive integrin-associated interactions, Vγ9Vδ2 T cells were pretreated with soluble RGD peptide (50 μM) before hydrogel stimulation. RGD pretreatment significantly suppressed SAM.1-induced upregulation of CD11c (41.0 ± 18.93% vs. 22.98 ± 8.08%; P < 0.05) and CD103 (8.17 ± 6.54% vs. 2.49 ± 0.29%; P < 0.05) (**Figures 6F, G**). In parallel, TNF-α expression was significantly reduced following RGD pretreatment (17,084 ± 2,980 vs.11,488 ± 1,783 MFI; P < 0.05),while CD25, IFN-γ, and perforin expression showed a downward trend, although not reaching statistical significance (**Figures 6H, I**). Importantly, RGD pretreatment did not affect integrin expression or effector molecule production in the Control, SAM.2, or SAM.3 groups, supporting the specificity of this functional inhibition in the context of SAM.1 stimulation. Collectively, these findings indicate that SAM.1 enhances Vγ9Vδ2 T-cell function in an integrin-associated and RGD-sensitive manner, supporting the involvement of integrin-mediated signaling. However, these data do not establish direct binding between SAM.1 and specific integrins, nor do they imply that CD11c or CD103 function as primary RGD-binding receptors in this context.

## Discussion

Melanoma remains a highly lethal solid tumor, with rising incidence and mortality worldwide. Conventional therapies, including surgery, radiotherapy, and chemotherapy, are effective for early-stage disease, yet advanced melanoma is prone to recurrence and metastasis, resulting in poor long-term outcomes (48). Immunotherapy, particularly immune checkpoint blockade (ICB) targeting PD-1/PD-L1 and CTLA-4, has transformed patient care. However, a substantial fraction of patients fails to respond or develops resistance, and immune-related adverse events are common (45), underscoring the need for alternative or complementary strategies. Adoptive cell therapy (ACT) offers such an avenue. CAR-T therapy has shown striking success in hematologic malignancies but faces major challenges in solid tumors, including limited trafficking, on-target/off-tumor toxicity, and dependence on patient T-cell quality (49).

γδ T cells are emerging as attractive candidates for cancer immunotherapy. This functionally distinct T-cell subset bridges innate and adaptive immunity, recognizing stress-induced ligands and phosphoantigens independently of MHC and mediating rapid cytotoxicity (50). Among them, the Vγ9Vδ2 subset, predominant in human peripheral blood, can release perforin and granzymes, induce Fas–FasL-mediated apoptosis, and secrete cytokines such as IFN-γ and TNF-α (51). Tumor infiltration by γδ T cells correlates with favorable prognosis across malignancies, and tissue-resident γδ T cells are linked to improved survival (18, 52). Clinically, Vγ9Vδ2 T cells can be expanded ex vivo using zoledronic acid, IL-2, IL-15, or vitamin C, with early trials in hepatocellular carcinoma and lung cancer showing survival benefits (28, 45). In melanoma, however, circulating Vγ9Vδ2 T cells are reduced and functionally impaired in advanced stages, recovering only partially after tumor resection (53), highlighting the need for strategies to enhance their antitumor efficacy.

Several barriers limit Vγ9Vδ2 T-cell therapy: short *in vivo* persistence, inefficient tumor infiltration, and susceptibility to immunosuppressive tumor microenvironments (TME) (21). Multiple infusions are often required, complicating clinical protocols. Hydrogel that delivering therapeutic cells, offer a promising solution, while various cytokines and complex preparation process are needed. Self-assembly materials, including hydrogels, constructed by peptides provide good manufacturing practices, biocompatibility and designable bioactivity. They possess the ability to tunable nanofiber networks, bioactivity, and biodegradability, supporting cell delivery, protecting against hostile TME cues, and enabling controlled local release (54–56). Rather than relying on canonical ligand motifs, such systems may engage immune cells through ECM-inspired, non-canonical cell–material interactions that promote integrin-associated signaling.

Here, we evaluated three self-assembly materials and identified SAM.1 (Nap-GFFF) as the most effective in potentiating Vγ9Vδ2 T-cell function. Structural analysis confirmed stable β-sheet formation and pronounced chirality, likely providing a supportive adhesive microenvironment. Functionally, SAM.1 enhanced activation markers (CD25, CD69, CD137) and cytokine secretion (IFN-γ, TNF-α, perforin) while maintaining >92% viability, with no cytotoxicity. Sustained release of encapsulated cells was observed, confirming suitability as a delivery scaffold (57).

Notably, SAM.1—but not SAM.2 or SAM.3—enhanced cytotoxicity against A375 melanoma cells *in vitro*. *In vivo*, peritumoral delivery of SAM.1-encapsulated Vγ9Vδ2 T cells suppressed tumor growth, increased serum IFN-γ, and promoted intratumoral infiltration. Pro-inflammatory cytokines, including IL-12p70, IL-18, and IL-33, were elevated, suggesting TME modulation. Local delivery of SAM.1-encapsulated Vγ9Vδ2 T cells resulted in robust antitumor efficacy, consistent with reports that localized hydrogel scaffolds can improve adoptive cell therapy performance (31). These results position SAM.1 as a dual-function platform: both a cell delivery vehicle and an active immunomodulator.

Mechanistically, SAM.1 activated the PI3K/AKT/mTOR pathway, evidenced by increased phosphorylation of AKT, mTOR, p70S6K, and 4E-BP1, whereas rapamycin inhibited these effects. This highlights mTOR signaling as essential for functional enhancement. While mTOR activation is typically achieved via soluble factors, our findings demonstrate that a peptide hydrogel can trigger this pathway through integrin-mediated cell–matrix interactions, introducing a novel paradigm in T-cell regulation. Importantly, although T cells are known to sense mechanical cues from their microenvironment, the differential activation observed with SAM.1 was not a generalized enhancement across all self-assembling materials, as SAM.2 and SAM.3 failed to induce comparable signaling or functional outcomes under identical preparation conditions. This selectivity supports a sequence-dependent biochemical mechanism, rather than nonspecific mechanical stimulation alone.

Metabolic reprogramming further contributed to SAM.1’s efficacy. Seahorse analysis showed enhanced oxidative phosphorylation and reduced glycolysis, shifting Vγ9Vδ2 T cells toward a more efficient metabolic state. This supports ATP generation, favors memory-like phenotypes, and may improve competition against tumor cells, counteracting the Warburg effect. Thus, SAM.1 strengthens γδ T-cell function by coupling integrin–PI3K/AKT/mTOR signaling with favorable metabolic reprogramming.

Integrins played a pivotal role in mediating SAM.1-induced activation. SAM.1 rapidly upregulated CD11c (αXβ2) and CD103 (αEβ7), and RGD peptide blockade attenuated integrin expression, TNF-α secretion, and activation. These results indicate that SAM.1 may engage RGD-recognizing integrins, triggering integrin clustering and downstream FAK–Src–PI3K cascades. However, we recognize that CD11c and CD103 are not classical RGD-binding integrins. Rather than directly mimicking RGD motifs, SAM.1 appears to interact with RGD-binding integrins (such as αvβ3 or αvβ5) indirectly, potentially through changes in the matrix environment or conformational effects. Transient integrin activation appears sufficient to induce durable functional and metabolic changes while avoiding exhaustion, and the three-dimensional nanofiber architecture enhances clustering beyond soluble ligands (58). Notably, integrin blockade blunted SAM.1-mediated activation despite preserved hydrogel structure, further supporting that biochemical ligand–receptor engagement, rather than bulk material properties, is required for the observed enhancement. We acknowledge that the precise integrin receptors responsible for sensing SAM.1 remain incompletely defined. Future studies should incorporate integrin-specific blocking antibodies (e.g., αvβ3, αvβ5, β1, or β2 family members), non-binding control peptides, and loss-of-function approaches to delineate receptor specificity. Structural modeling or biophysical analyses, such as integrin-binding assays or molecular docking, may further clarify whether SAM.1 induces conformational states permissive for integrin activation. These studies will be critical to distinguish direct ligand–receptor interactions from indirect, architecture-driven integrin engagement.

Overall, SAM.1 addresses key barriers in γδ T-cell therapy, including persistence, infiltration, and functional suppression. Its self-assembly, low cost, and biocompatibility enhance translational potential. Limitations include use of a single melanoma cell line (A375), immunodeficient mouse models, and incomplete understanding of precise peptide–integrin interactions. Future studies should explore other melanoma subtypes, evaluate immunocompetent or humanized models, and elucidate binding kinetics. Comprehensive assessment of degradation, biocompatibility, and biodistribution is needed prior to clinical translation. In addition, although all hydrogels were prepared at identical peptide concentrations and under comparable self-assembly conditions, we did not directly assess their rheological properties. Therefore, we cannot fully exclude the possibility that subtle differences in stiffness may contribute to T-cell activation. Given the known mechanosensitivity of T cells, future studies incorporating rheological characterization and experimental decoupling of mechanical versus biochemical cues will be important to further clarify the relative contributions of hydrogel stiffness and peptide sequence.

Looking ahead, SAM.1’s tunable properties allow optimization for different tumor types or delivery routes. Combination therapies with checkpoint inhibitors or costimulatory cytokines may further enhance efficacy. Given the central role of integrin–mTOR–metabolism signaling, this approach may extend to other immune effectors, including CAR-T or NK cells, broadening therapeutic applications. Collectively, these findings provide a strong rationale for early-phase clinical trials to evaluate safety and efficacy in patients.

## Conclusion

**Figure 7:**
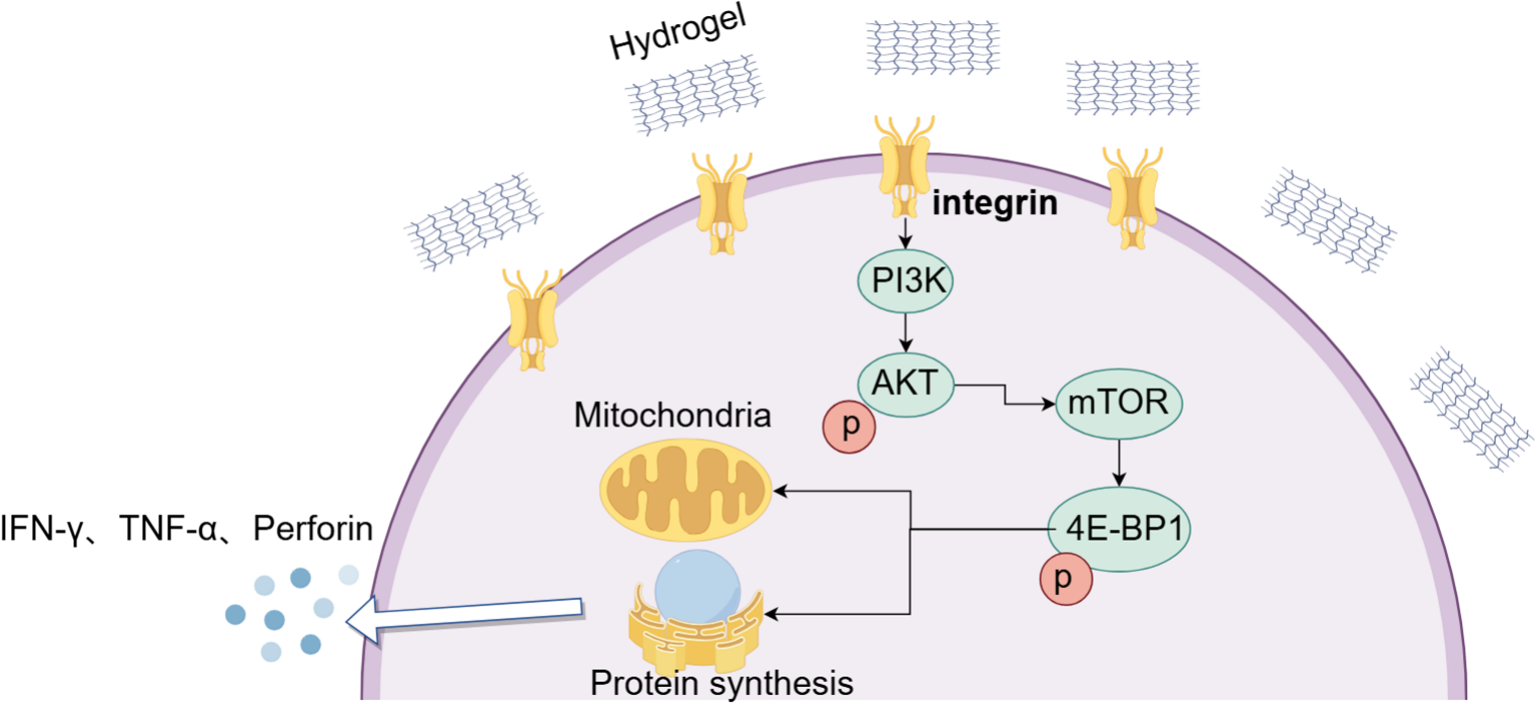
Proposed model of SAM.1 hydrogel–mediated enhancement of Vγ9Vδ2 T-cell function. SAM.1 hydrogel engages integrin-associated, RGD-sensitive signaling on Vγ9Vδ2 T cells to activate PI3K/AKT/mTOR pathways. This activation improves mitochondrial function and shifts metabolism toward oxidative phosphorylation, which enhances protein synthesis and effector function. As a result, Vγ9Vδ2 T cells exhibit increased activation and elevated secretion of IFN-γ, TNF-α, and perforin, thereby strengthening their antitumor capacity.

In summary, we identified peptide hydrogel as a safe and effective platform to potentiate Vγ9Vδ2 T-cell immunotherapy. SAM.1 enhanced T-cell activation, cytokine secretion, and cytotoxicity *in vitro*, and improved tumor infiltration and antitumor efficacy *in vivo*. Mechanistically, our data support a model in which SAM.1 promotes integrin-associated and RGD-sensitive signaling, accompanied by activation of the PI3K/AKT/mTOR signaling and reprogram metabolism toward oxidative phosphorylation, thereby strengthening T-cell persistence and effector function (**Figure 7**). While the precise molecular interactions between SAM.1 and integrins remain to be fully defined, these findings indicate that modulation of integrin-related signaling is a key component of SAM.1-mediated T-cell enhancement. Collectively, our study highlight SAM.1 as a clinically translatable strategy that integrates targeted delivery with intrinsic activation, offering a dual approach to improve γδ T-cell therapy for melanoma and potentially other solid tumors.

## Data Availability

The raw data supporting the conclusions of this article will be made available by the authors, without undue reservation.
