# Supplementary material for: Peptide hydrogel boosts the cytotoxic and metabolic fitness of Vγ9Vδ2 T cells in melanoma immunotherapy
Source: Front Immunol. 2026 Mar 20;17:1793631. doi: 10.3389/fimmu.2026.1793631 (PMC13047159; doi:10.3389/fimmu.2026.1793631)
Supplement: Supplementary file 1 [file DataSheet1.pdf]

Supplementary Material

1 Supplementary Figures and Tables

1.1 Supplementary Figure S1. Flow cytometry gating strategies for cytotoxicity, activation, cytokine production, and integrin expression in Vγ9Vδ2 T cells.

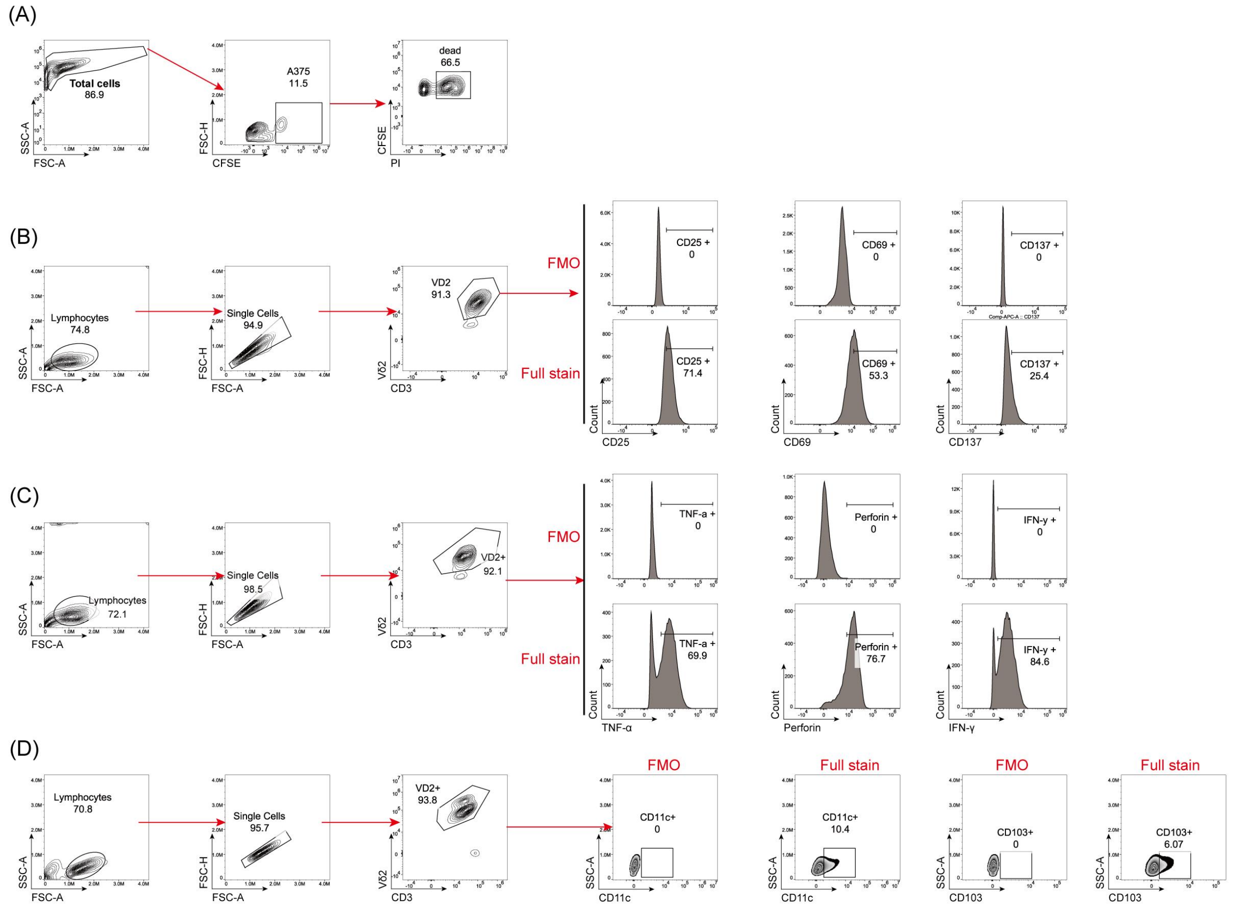

(A) Gating strategy for cytotoxicity assays. Total cells were first gated by FSC/SSC to exclude debris, followed by identification of CFSE-labeled A375 target cells. Dead target cells were defined as $CFSE^{+}PI^{+}$ events, and Cytotoxicity was calculated as: Cytotoxicity (%) =[(% of dead target cells – % of spontaneous death)/(100 – % of spontaneous death)] × 100%. (B-C) Gating strategy for activation marker and intracellular effector molecule analysis. Lymphocytes were gated by FSC/SSC, followed by singlet discrimination and identification of Vγ9Vδ2 T cells ($CD3^{+}V\delta2^{+}$). Activation markers (CD25, CD69, CD137) and effector molecules (IFN-γ, TNF-α, and perforin) were analyzed within the Vγ9Vδ2 T-cell gate. Fluorescence minus one (FMO) controls were used to define positive gates. (D) Gating strategy for integrin-associated surface markers analysis. After identification of Vγ9Vδ2 T cells, expression of CD11c (ITGAX) and CD103 (ITGAE) was quantified. Representative fluorescence minus one (FMO) controls are shown for low-abundance markers to define positive

gates. For markers assessed in multiple panels, representative full-stain/FMO plots are shown once to avoid redundancy.

1.2 Supplementary Table 1. Reagents and chemicals used in this study.

Reagent	Company	Country	Catalog No.
RPMI-1640 medium	Gibco	USA	11875-093
DMEM medium	Gibco	USA	11965-092
Ficoll-Paque PLUS	GE Healthcare	USA	17-1440-03
Red blood cell lysis buffer	Tiangen Biotech	China	RT122
Recombinant human IL-2	Beijing Sihuan Biotech	China	/
FOXP3/Transcription Factor Staining Buffer Set	Invitrogen (eBioscience)	USA	00-5523-00
PMA (Phorbol 12-myristate 13-acetate)	Sigma-Aldrich	Germany	P8139
Ionomycin	Sigma-Aldrich	Germany	I0634
GolgiPlug/GolgiStop	BD Biosciences	USA	555029/554724
Annexin V-FITC/PI Apoptosis Detection Kit	BioLegend	USA	640914
Human Inflammation Panel 1 (13-plex) with Filter Plate	BioLegend	USA	740809
SYBR Premix Ex Taq Kit	Takara	Japan	RR420A

Supplementary Table 1. Reagents and chemicals used in this study.
List of reagents, chemicals, and culture media used in this study, including supplier, country of origin, and catalog numbers.

1.3 Supplementary Table 2. Antibodies for flow cytometry and Western blotting.

Antibody	Company	Country	Catalog No.	Clone
mTOR Substrates Antibody Sampler Kit (p-mTOR, p-S6K, p-4EBP1)	Cell Signaling Technology	USA	9964	/
PI3K antibody	Cell Signaling Technology	USA	4255	/
AKT/p-AKT antibody	Cell Signaling Technology	USA	4691/4060	/
FITC anti-human CD3	BD Biosciences	USA	555339	UCHT1
PE anti-human Vδ2	BioLegend	USA	555718	B6
Spark Blue 550 anti-human CD3	BioLegend	USA	317328	OKT3
FITC anti-human Vδ2	BioLegend	USA	331404	B6
BV480 anti-human CD69	BD Biosciences	USA	562884	FN50
BV421 anti-human Perforin	BD Biosciences	USA	563762	dG9
APC anti-human CD137 (4-1BB)	BioLegend	USA	309810	4B4-1
PE-Fire 640 anti-human CD25	BioLegend	USA	356118	M-A251
PE-Cy7 anti-human IFN-γ	BD Biosciences	USA	557643	B27
PerCP-Cy5.5 anti-human TNF-α	eBioscience	USA	45-7349-42	MAb11
PE anti-human CD107a (LAMP-1)	BioLegend	USA	328608	H4A3
Alexa Fluor 647 anti-human CD11c	BioLegend	USA	337229	3.9

**Antibody**	**Company**	**Country**	**Catalog No.**	**Clone**
APC-Cy7 anti-human CD103	BioLegend	USA	350227	Ber-ACT8

Supplementary Table 2. Antibodies for flow cytometry and Western blotting.
Antibodies used for flow cytometry and Western blotting, including fluorochrome conjugates, clones, suppliers, and catalog numbers.

1.4 Supplementary Table 3. Primer sequences for qPCR (TRGV9, PTPRC).

Primer name	Sequence (5′→3′)
TRGV9-F	5'-GGA TCC TCA GCA AGC AAA GA-3'
TRGV9-R	5'-TGT CAG GGT GTC CAG GTT CT-3'
PTCRC-F	5'-CTG GAG GCT GAA CAT GGA G-3'
PTCRC-R	5'-TGA GCA GCA GCA TCA TCA TC-3'

Supplementary Table 3. Primer sequences for qPCR.
Primer sequences used for quantitative PCR (qPCR) analysis of human TRGV9 and PTPRC (CD45) gene expression.

1.5 Supplementary Table 4. Animal experimental design (groups, n, treatment schedule).

Group	Treatment (weekly × 4)	Route of administration	Cell dose per mouse	N (number of mice)
Control	PBS	Peritumoral (s.c.)	/	5
γδT (s.c.)	Vγ9Vδ2 T cells	Peritumoral (s.c.)	1×10^7 cells	5
SAM.1 + γδT (i.v.)	SAM.1-encapsulated Vγ9Vδ2 T cells	Intravenous ((i.v.)	1×10^7 cells	5

SAM.1 + γδT (s.c.)	SAM.1-encapsulated Vγ9Vδ2 T cells	Peritumoral (s.c.)	1×10^7 cells	5

Supplementary Table 4. Animal experimental design for the B-NDG melanoma xenograft model.
Detailed experimental design of the animal study, including group assignments, treatment regimens, administration routes, cell doses, and number of mice per group.
